# Economic burden to primary informal caregivers of hospitalized older adults in Mexico: a cohort study

**DOI:** 10.1186/1472-6963-13-51

**Published:** 2013-02-08

**Authors:** Mariana López-Ortega, Carmen García-Peña, Víctor Granados-García, José Juan García-González, Mario Ulises Pérez-Zepeda

**Affiliations:** 1 Instituto Nacional de Geriatría, Periférico Sur 2767, Colonia San Jerónimo Lídice, Delegación Magdalena Contreras, México D.F, Mexico; 2 Unidad de Investigación Epidemiológica y en Servicios de Salud, Area de Envejecimiento, Centro Médico Nacional Siglo XXI; Instituto Mexicano del Seguro Social, México, D.F, Mexico; 3 Hospital General Regional No. 1, Instituto Mexicano del Seguro Social, Querétaro, Querétaro, Mexico

**Keywords:** Elderly, Caregiver expenses, Acute geriatric care, Out of pocket expenses

## Abstract

**Background:**

The burden of out of pocket spending for the Mexican population is high compared to other countries. Even patients insured by social security institutions have to face the cost of health goods, services or nonmedical expenses related to their illness. Primary caregivers, in addition, experience losses in productivity by taking up responsibilities in care giving activities. This situation represents a mayor economic burden in an acute care setting for elderly population. There is evidence that specialized geriatric services could represent lower overall costs in these circumstances and could help reduce these burdens.

The aim of this study was to investigate economic burden differences in caregivers of elderly patients comparing two acute care services (Geriatric and Internal Medicine). Specifically, economic costs associated with hospitalization of older adults in these two settings by evaluating health care related out of pocket expenditures (OOPE), non-medical OOPE and indirect costs.

**Methods:**

A comparative analysis of direct and indirect costs in hospitalised elderly patients (60-year or older) and their primary informal caregivers in two health care settings, using a prospective cohort was performed. Economic burden was measured by out of pocket expenses and indirect costs (productivity lost) due to care giving activities. The analysis included a two-part model, the first one allowing the estimation of the probability of observing any health care related and non-medical OOPE; and the second one, the positive observations or expenditures.

**Results:**

A total of 210 subjects were followed during their hospital stay. Of the total number of subjects 95% reported at least one non-medical OOPE, being daily transportation the most common expense. Regarding medical OOPE, medicines were the most common expense, and the mean numbers of days without income were 4.12 days. Both OOPE and indirect costs were significantly different between type of services, with less overall economic burden to the caregivers of elderly hospitalized in the geriatric unit. The final model showed that type of service and satisfaction had the largest coefficients (-0.68 and 0.662 respectively, p<0.001).

**Conclusions:**

This study allowed us to identify associated factors of economic burden in elderly hospitalized in acute care units. It opens as well, an issue that should not be overlooked in framing public policies regarding elderly health care.

## Background

With the almost universal ageing process and increasing morbidity, changes and great challenges to health and social care systems are expected. In Mexico, the population structure by age is changing quickly and will pose high burden to health, social care, and social security systems [1]. In addition, the Mexican Health System has been highly segmented since its conception, causing large disparities in access and a lack of response to health needs of older adults. Social security institutions provide health and social services to those with a formal employment status, and their eligibility for pensions and retirement benefits will depend on past participation in formal employment; including the Mexican Institute of Social Security (IMSS) for those working in the formal private sector, the Institute of Security and Social Services for Government Employees (ISSSTE) for federal level and state level government employees, and institutions covering the Navy, Armed Forces, and Mexican Petroleum Company.

The main social security provider is the IMSS covering approximately 40% of the total population in Mexico, through affiliated individuals and their dependents. Those affiliated to IMSS have access to a more comprehensive health care system as well as to economic benefits, compared with members of other social security institutions, the uninsured, and those who access services through the Ministry of Health [2].

In a gross division, health care includes ambulatory and hospital care. Hospital care is centred in acute care for emergency events or chronic disease complications or exacerbations. Long-term care is not available at IMSS or at any other health system in Mexico. Elders that require hospitalization are mostly cared at Internal Medicine Wards (IMW) since specialized geriatric care units, called Geriatric Evaluation and Management Units (GEMU), are scarce. In the IMSS health system, there is only one specialized unit in elderly care, located in Mexico City.

Mexican families from all socioeconomic levels and regardless of their public insurance condition have to face health care related costs, and it has been estimated that up to 50% of health care related expenditures are financed directly by households through out of pocket expenditures (OOPE). This fact has been described as a clear sign of prevalent high inequalities in health care financing in the country [3-5]. Health care related OOPE have been defined as those expenses for prescribed medicines, consultations and laboratory tests, as well as payments for conditions or treatments not included in health insurance plans (fees and co-payments). On the other hand, non-medical OOPE include a much wider array of categories such as transport to the point of service, parking fees, special food, lodging, cost of having to pay for additional caregivers, personal care items, among others. Moreover, another source of expenses are indirect costs, which include lost income due to unpaid work and/or reduced hours of work [6]. In Mexico, patients and their caregivers, despite of their insurance status, have to incur in these health care and non-medical OOPE. Non-medical OOPE have been noted to be higher in acute care of elderly settings due to higher number of days of hospitalization, higher intensity of care needed, among other reasons [7-9].

There is conflicting evidence regarding the impact of overall costs related to acute care for elderly in hospitals and its difference in economic burden, comparing between an IMW and a GEMU. Moreover, usually the main focus has been on direct health care costs to the institution providing services, rather than in quantifying the burden posed to the family or other informal care givers due to indirect costs, health care related, and non-medical OOPE (overall economic burden) [10,11].

Accordingly, the aim of this study was to investigate possible differences between a GEMU and an IMW at the IMSS, besides the favourable clinical outcomes already published by our group [12]. Specifically, to investigate issues related to economic costs associated with hospitalization of older adults in these two settings by evaluating economic burden (health care related and non-medical OOPE, as well as indirect costs) of patients and their caregivers treated at a GEMU and IMW.

## Methods

### Data

A comparative analysis of direct and indirect costs in hospitalised elderly patients and their primary informal caregivers at a GEMU and an IMW was undertaken with data from a cohort study on health outcomes and costs in these two services in a hospital setting at IMSS. The design of that study was a prospective cohort of matched triplets—by age, sex and main diagnosis at admission; assessing a set of health outcomes such as falls, pressure sores, delirium, and functional decline at discharge (GEMU vs. IMW). Methods, description of the models and results of the study are reported elsewhere [12].

Briefly, over a two-year period (2007–2009) patients aged 60 years and older were recruited from among those admitted to either the GEMU or the IMW. Inclusion criteria were the presence of at least one geriatric problem (falls, slow walking speed, tiredness, sorrow, depression, memory deficit, difficulty with instrumental activities, and bathing), as assessed at the first visit after admission. Patients with altered consciousness or not able to communicate, referred from the Intensive Care Unit, and under mechanical ventilation or parenteral nutrition were excluded. For each patient selected for the GEMU, two patients were matched (by main diagnosis, age and gender) in the IMW group. According to van Craen, a difference of 13% in functional decline between groups was expected (favoring GEMU) [13] and it was determined that a minimum of 70 study subjects in the GEM group and 140 subjects in the IMW group was needed with a beta of 80% and a significance level of 0.05. Due to the fact that there are no reports on this issue, we used this sample to detect differences in non-medical OOPE between the study subjects. The data set included relevant information on patient’s socio-demographic characteristics and health status, as well as some information on their primary caregiver (defined as the person who spent most of the time with the elder during their hospital stay, and knew most of the information regarding the health status of the patient) [14]. Trained and standardized nurses collected the data at the hospital in a daily basis.

### Definition of health care related OOPE, non-medical OOPE and indirect costs

Economic burden to the primary informal caregiver was defined as a continuous variable representing the sum of all expenses patients and their caregivers made during the hospital stay. This variable included health care related OOPE: medicines and other medical equipment, or paying for a caregiver; and non-medical OOPE: food and transport. In addition, forgone hours of work and lost income due to care giving were also investigated and defined as indirect costs.

The measurement to determine health care related and non-medical OOPE as well as indirect costs was based on self-reported information from patients and their primary informal caregiver about different domains related to the hospitalization event. Specifically, detailed questions on common health care related OOPE such as medicines and mobility aids, as well as personal care supplies, and non-medical OOPE such as transportation costs and food. Indirect costs included self-reported data on employment or work condition, lost working days or reduced hours of work due to care giving and related activities, as well as self-reported lost income both from formal employment and/or any other activity (such as self-employment) that the caregiver performs on a regular basis.

Given the lack of Long-term care services and a Long-term care labour market in Mexico, as well as the information collected in the survey, the method used to value indirect costs was the human capital approach. This method takes the caregiver’s perspective into account and takes into account all hours not worked and income forgone due to caregiving and related activities (transportation, buying devices, etc.). In order to reflect working conditions in Mexico and the information captured in the questionnaire, income lost included all self-reported forgone income due to caregiving and related activities, independently of the main occupation or activity the caregiver had. Productivity loss due to forgone income of caregivers was estimated as follows: number of working days missed (whether from formal employment or main income-generating activity) due to caregiving, multiplied by daily income, resulting in the total income lost. For presentation purposes all monetary values are expressed in US dollars (US$) with an official Central Bank exchange rate of 11.68 Mexican pesos (MXN$) per dollar (June 2011).

Given the setting in this cohort study and the high training of the interviewers, the recall method was considered as the optimal way to gather information on expenditures, along with the rest of self-reported information on the patient and the care provided during the hospital stay. This adds to the scarce studies in the country that captures expenditures in such health care settings, as well as productivity loss due to care giving activities.

### Variables and statistical analysis

Analysis included deceased persons during hospitalization. A descriptive exploration of overall OOPE and indirect costs in the sample and an investigation of possible differences between different groups within the sample were performed. To estimate central tendency measures, deviation, and variance, one and two-way analyses were undertaken. Comparison of means between type of service and the rest of the variables was carried out in order to compare differences between groups and to test their significance using Student’s *t*-test, with the exception of number of caregivers where the chi-squared test was used.

In a second step, regression analyses were conducted in order to identify independent predictors of overall OOPE and indirect costs. A skewed distribution was expected, with a long right tail, thus, a model recognizing that distribution and less sensitive to right tail issues was selected [15]. A two-part model was estimated by means of a probit model for the probability of observing a positive value of overall OOPE (y), along with an OLS on the subsample of positive observations (level of expenditures), conditional on y>0. In order to correct for possible bias due to skewed data, for the second equation a linear model on the log scale for positive expenses was used [16]. Post estimation tests to evaluate the fit of the models included the Hosmer-Lemeshow goodness of fit and the Brauch-Pagan/Cook-Weisberg test for normality of residuals. The analysis adopted the societal perspective given it represents the most comprehensive approach within the information and data available from the study, which includes costs paid by patients and their primary informal caregivers such as health care relate and non-medical OOPE, as well as indirect costs associated to these hospitalization events.

Previous studies have found a strong association between socio demographic characteristics of the patient and comorbidity and OOPE. In addition, service related factors have also been linked to OOPE [17-19]. In order to see if same variables could have an effect in our population study, OOPE was modelled as a function of the following variables: sex, age, activities of daily living (ADL) at admission, comorbidity, acute illness severity, cognitive status, and hospital length of stay. In order to reflect service characteristics we included length of stay, type of service (GEMUs or IMW) and satisfaction. Within the scope of our study, we hypothesise that higher comorbidity, longer hospital stays, a higher severity in the acute illness, cognitive impairment, more depressive symptoms and loss of functional ability result in higher OOPE and that a lower satisfaction with the formal services provided by the hospital results in a higher perceived need for informal care and related OOPE. In general terms, each of these variables could impact in one or more of the following causes of an OOPE or indirect cost: use of more drugs, use of more specialized studies, and the need for the continuous presence of the caregiver in the hospital (details in Additional file 1: Annex 1).

ADL were measured using the *Barthel Index*, and the variable in the model was coded as continuous (0 to 100), being the higher the score the more functional the subject [20]. Comorbidity was measured using the *Charlson Index*, which is a summary measure that assesses the weight of chronic diseases on survival, with a score ranging from 0 to 20, the latter corresponding to the most comorbid subject [21]. Cognitive function was measured using the *Mini Mental State Examination*; dichotomized as 0 for those with normal cognitive function and 1 for those who present cognitive impairment [22]. Finally, the *Acute Physiology and Chronic Health Evaluation* II (APACHE II) score was included in the model as an assessment of hospital mortality risk of acutely ill patients [23]. For service characteristics, the models include a dummy variable representing the type of service (1=GEMU); and a variable for satisfaction derived from the Client Satisfaction Questionnaire (CSQ 8) was asked “How would you rate the quality of the care you received during this hospitalization?” with two possible mutually exclusive answers:well (satisfied) and average/bad (not satisfied) [24]. Additional file 1: Annex 1 includes a glossary with a more detailed description of these health related performance measures, their scores and interpretations, as well as the effect we hypothesise they will have in the model estimations. All estimations were done using STATA© version 11.

### Ethical considerations

National Scientific Research Commission at IMSS approved the study; with the following registration number: 2005-785-170. Also, research was carried out in compliance with the latest version of the Helsinki Declaration.

## Results

### Descriptive statistics

The final sample in the study included 70 matched older adults admitted to a GEMU and 140 older adults admitted to an IMW for a total of 210 older adults grouped in 70-matched triplets. In-hospital mortality was 6.66% (14 subjects). Description of the sample is presented in Table 1. Baseline characteristics of these subjects were not significantly different from those who survived. Patients in both groups (GEMU vs. IMW) had a similar age distribution, with a combined average age of 72.5 years.

**Table 1:** Definition of variables and main statistics, stratified by service (IMW vs GEMU)

**Variables**	**Description**	**IMW (n=140)**	**GEMU (n=70)**
***Expenditures***			
Total overall OOPE	Mean of total expenditures (±SD)	174.0 (251.15)	67* (66.52)
Binary overall OOPE	Variable indicating patient/caregiver incurred in expenses; percentage; 1=Yes	83.70	84.06
***Older adult***			
Age	Mean age of hospitalised patient (±SD)	72.3 (7.7)	72.3 (7.2)
Male	Gender of hospitalised patient percentage; 1=male	44.12	41.43
Acute illness severity	APACHE II score (±SD)	11.4 (5.1)	10.62 (4.6)
Comorbidity	Charlson Index score (±SD)	8.57 (3.05)	8.21 (2.78)
ADL	Barthel Index (±SD)	85 (23.59)	89.3 (19.7)
Cognitive status	MMSE score (0–30) (±SD)	19.7 (6.5)	21.0 (6.4)
Cognitive decline	Diagnosis of cognitive decline percentage; 1=cognitive decline	47.8	35.1
***Services***			
Satisfaction	Satisfaction with care/service received percentage; 1=average/bad⌐ .	22.4	6.5*
Length of stay	Mean of number of days in hospital (±SD)	9.26 (6.2)	9.87 (10.01)
***Caregivers***			
Gender	Gender of caregiver (1=male)	11.4	23.4**
Age	Mean age of caregiver (±SD)	47.4 (11.78)	46.87 (13.72)
***No. caregivers/patient***	Number of caregivers by patient throughout the stay; percentage;		
0	no caregiver	16.18	8.6**
1	one caregiver	43.38	68.6
2	two caregivers	27.94	17.1
3	three caregivers	9.56	5.7
4	four caregivers	2.21	
5	five caregivers	0.74	
Days without income	Mean of days of lost income due to caregiving (±SD)	4.04 (8.43)	4.20 (9.16)
Mean caregiver income	Mean monthly income of caregiver (±SD)	404.76 (768.32)	216.25 (169.37)

*Notes:* IMW=internal medicine ward; GEMU=geriatric evaluation and management unit; OOPE=out-of-pocket expenditures; SD=standard deviation; ADL=activities of daily living; APACHE II=Acute Physiology and Chronic Health Evaluation II; MMSE=Mini-Mental Status Examination.

Amounts are in US Dollars, exchange rate of 11.68 Mexican pesos for each $1.00 US Dollar at June 2011.

⌐ Reference category=good.

* p value ≤ 0.005; ** p value ≤ 0.05.

Of the 196 older adult patients who finished the study, 28 (13.33%) did not receive care or support from an informal caregiver (family member, friends, etc.) during their hospital stay. The majority (51.9%) reported having one main informal caregiver, while almost a fourth (24.3%) received support from 2 caregivers. The total number of caregivers in the sample was 178 subjects. Of them, 84% were women (n=150), and their mean age was 47.3 years (SD±12.6) (Table 1).

Results of descriptive analysis showed that characteristics of patients in both services were very similar at admission, with no statistically significance between them (Table 1). Regarding the caregiver characteristics, there was a higher percentage of male caregivers for patients at the GEMU with a significant difference (p<0.05). There was also a statistically significant difference between the two types of services in the number of informal caregivers per patient, with patients at the IMW requiring up to five caregivers. With respect of health services utilization, Table 1 shows that length of stay was similar between the two types of services with 9.26 days (SD ±6.2) for the IMW and 9.55 (SD ±10.1) at the GEMU. In contrast, difference in user satisfaction with care received was statistically significant between the services (p<0.01) with 22.4% of patients at IMW perceiving the care as average/bad against only 6.5% of patients at GEMU having this perception. Regarding differences in overall OOPE, tests showed significant differences between the two types of services (p<0.001), with lower overall OOPE in the GEMU.

Of the total number of caregivers (n=178), only 5% reported no overall OOPE. The descriptive analysis of overall OOPE showed that, for the remaining 95% who did spend, the average daily transportation cost was $4.6 USD (SD ±15.4). Caregivers’ expenses at IMW were higher than those at the GEMU ($6.2 USD, SD±18.7 versus $2.5 USD, SD±2.9 respectively) (Table 2). With the exception of OOPE in medicines, the remaining items of expenditure had a significant difference between overall OOPE in the two services (p<0.05).

**Table 2:** Overall OOPE related to hospitalization of an older adult, stratified by service (IMW vs GEMU)

**Type of expenditure**	**IMW Mean (±SD)**	**GEMU Mean (±SD)**
Medicines	3.1 (18.1)	0.21 (1.6)
Other medical supplies and personal care^†^	2.9 (2.5)	0
Transportation^†^	6.2 (18.7)	2.5 (2.9)
Food^†^	6.6 (16.5)	3.1 (3.8)

*Notes:* IMW=internal medicine ward; GEMU=geriatric evaluation and management unit; SD=standard deviation.

Amounts are in US Dollars, exchange rate of 11.68 Mexican pesos for each 1.00 US$ (June 2011).

†Significant at p ≤ 0.05

### OOPE

In both services, IMW and GEMU, OOPE in food and transportation represented the highest expenditures. Comparing the two services, food related expenses at GEMU were $3.1USD (SD ±3.8), representing almost 50% less than those at IMW where the average was $6.6 USD (SD ±16.5) per day. With respect to medicine expenditures, 14 caregivers reported OOPE on up to three different medicines. Of these, 93% were caring for a patient admitted to an IMW. None of the carers reported OOPE in laboratory tests or studies.

Finally, OOPE to pay for a formal caregiver was analysed. A total of three remunerated caregivers, all in the IMW were hired to take up caring activities, for an average payment of $23.5 USD (SD ±3.0) per day, for a total average-length of stay payment of $122.0 USD (SD ±99.8).

### Indirect costs

Regarding indirect costs derived from absent days from work and income lost during care giving at hospital for elderly patients; 57% of all carers (n=104) declared having to suspend their main occupation or work in order to care for the hospitalized older adult. Patients admitted at IMW received more informal care from family and friends, with 72% (n=75) of the total number of caregivers. The average income for caregivers (n=123) was $350 USD, with a difference in average income of caregivers between IMW ($405.4 USD; SD ±751.9) and GEMU ($216.2 USD; SD ±169.3). For those caregivers that had to stop their main occupation or work, the average number of days not receiving an income was 4.3 (SD ±8.8). Overall, productivity lost was on average $99 USD (SD ±661.4; 95% CI -19.2- 216.9). While income lost by caregivers at IMW was on average $131.3 (SD ±785.2; 95% CI -36 -298.7), for GEMU the average was $20.5 USD (SD±35.6; 95% CI 8.4-32.5).

### Factors related to OOPE

Results of the two-part model are presented in Table 3. For the full sample, being male, cognitive impairment and a high Charlson Index were associated with a higher probability of having any OOPE in the first part of the model, although only the coefficient for being male was statistically significant (*p*<0.05). Length of stay was also significant (p<0.05) in determining the probability of having OOPE.

**Table 3:** Two-part model of OOPE

**Variables**	**First-part (n=182)**		**Ln of OOPE (n=159)**	
***Overall OOPE total***	**Coefficient**	**(SE)**	**Coefficient**	**(SE)**
Service	-0.065	(0.268)	-0.680***	(0.201)
Satisfaction	-0.256	(0.343)	0.662**	(0.263)
Male	0.472*	(0.270)	0.213	(0.190)
Age	-0.012	(0.017)	0.025*	(0.012)
MMSE	0.323	(0.292)	0.366	(0.201)
ADL	-0.004	(0.006)	0.001	(0.004)
APACHE II	-0.026	(0.027)	0.002	(0.018)
Charlson Index	0.005	(0.044)	-0.060	(0.032)
Length of stay	0.078*	(0.034)	0.011	(0.017)
Constant	1.812	(1596)	2.753	(1.137)

*Notes:* OOPE=out-of-pocket expenditures; SE=standard error; MMSE=Mini-Mental Status Examination; ADL=activities of daily living; APACHE II=Acute Physiology and Chronic Health Evaluation II.

* p value ≤ 0.05; ** p value ≤ 0.01; *** p value ≤ 0.001.

In the OLS equation (conditional on having any OOPE), there was an association between type of service, satisfaction, and age with level of OOPE. Type of service presents the largest coefficient, showing a negative relation with overall OOPE. Compared to IMW, those at the GEMU had significant less expenses and this was statistically significant (p=0.001). Satisfaction with services also resulted to be significantly associated with OOPE (p≤0.01). Regarding health conditions and comorbidity, cognitive function seems to be positively associated both with the probability of OOPE and the level of expenditures, with high coefficients in both cases, although the coefficients were not statistically significant. Finally, age is positively associated with level of expenditures and the coefficient significant (p≤0.05) (Table 3). The fit of the model is considered adequate with *χ*^*2*^*=* 2.89 on 8 degrees of freedom and p=0.94. In addition, there seems to be no evidence of heteroscedasticity (*χ*^*2*^*=*0.97, p=0.32).

## Discussion

In this study, we investigated the economic burden associated with hospitalisation for acute care of older adults, by evaluating health care related and non-medical OOPE in two care settings: a GEMU and IMW at IMSS. In contrast to estimating health care system costs it rather quantifies the costs that an individual patient, and their families or primary caregivers pay when faced with such events, through overall OOPE. It also investigates indirect costs by caregivers when they have to stop or reduce work hours, or hours spent in other income-generating activities such as informal and self-employment, due to their care giving responsibilities. As stated previously, these results add to a previous report in which geriatric outcomes were more favourable in the GEMU compared to IMW [12]. Moreover, it takes into account indirect costs of acute care of elderly, rather than analysing only direct health costs; this opens a new approach to cost effectiveness analyses using a more holistic picture of the actual costs and the potential benefit of a GEMU [10]. The results of our analyses suggest that overall OOPE by caregivers of a patient at IMW are significantly higher than for those caring a patient at GEMU with figures at IMW that are at least double of those at GEMU. This is the case for transportation costs as well as for food, non-medical OOPE. Mediators of these differences are related to the early detection of high-risk elderly in the GEMU and a closer approach with the caregivers, which in turn are more satisfied and confident and with less perceived need to remain in the hospital due to the coordination between health professionals and caregivers in GEMU settings [13,25].

Even when overall OOPE are common when a family member is hospitalized, policy makers need to understand the economic burden these expenses represent for the household; future research is needed to approach these issues. Individual social costs faced by patients and their families due to hospitalization or other health care use are seldom taken into account, and when services or health care reforms are proposed, usually the attention is centred in the costs incurred by the health care delivery institutions, and little on the costs incurred by patients, families and primary caregivers, including health care related and non-medical OOPE, as well as indirect costs [2].

Taking into account that for the year 2011 the daily minimum wage in Mexico City was $5.12 US dollars [26] it is clear that non-medical OOPE can represent high expenditures, with care givers spending more than the daily minimum wage, in particular those caring for a patient at the IMW. The results show the high economic impact non-medical OOPE can have to households in Mexico, and in particular households with older adults.

With respect to the income reported by these care givers, comparing their average daily expenditures reported with their average daily reported income, OOPE represent approximately 35% and 19% of daily income for care givers at IMW and GEMU respectively. Even when analyses of catastrophic expenditures usually include OOPE with respect to full household expenditures, we can assume that these older adult households do not have many additional income sources, and thus these representing considerable expenditures.

Within the economic evaluation literature, the fact that bias may arise in using recall methods and self-reported data to gather expenditure information has been widely discussed. While some studies favour the use of diaries for the patients and their caregivers to fill out detailed information on their expenditures, and others have relied on direct recall of expenditures, there is no consistency in the results, and therefore no consensus on the optimal method. For example, a recent review by the World Health Organization [27] to investigate current evidence on measurement errors in self-reported household expenditure and health expenditure finds inconsistent results in the estimation of total expenditure by reporting method, number of questions or items included, the use of bounded or unbounded interviews, the type of goods the studies included, among other issues. Some of the results of the review show how even when diaries are considered to retrieve more accurate data, at least one fourth were filled at moment of pickup and therefore based on recall, they tended to produce loss of interest in their completion as time from event passed. Regarding the results, while some diaries produced lower expenditures versus face to face interviews, others report higher estimates in a diaries compared to surveys. Given that we do not have similar studies in the country to compare our results, it is hard to assert the exact accuracy of the self-reported data. Due to the highly assumed responsibility of caring, it is likely that carers oversee reporting small payments or related errands, and these may underestimate the current results. In addition, in calculating productivity loss, income loss is taken into account but we had no information on the total time the care giver spent taking up these caring activities, and therefore we could not measure other opportunity costs like loss of time forgone in leisure activities, carrying out other care-related errands, or specific care giver burden. These again, could yield an under estimation of the full costs of informal care giving for older adults in Mexico and their opportunity costs.

On the other hand, when assessing productivity losses, several methods can be used such as the willingness-to-pay approach; the human capital approach and the friction cost approach, being the latter the two most frequently used methods. In this context, the human capital method takes the caregiver’s perspective into account and takes into account all hours not worked and income forgone due to care giving and related activities (transportation, buying devices, etc.), taking into account potential and actual losses. The friction cost approach method on the other hand, aims at estimating actual production lost or costs forgone until that worker is replaced. There is no consensus on which of these methods is best with the human capital model often noted as overestimating actual productivity loss and the friction cost method being difficult to implement given its requirement of detailed data on labour market conditions [28,29].

Future studies have to be generated in order to explore non-medical OOPE with respect to total household expenditures and investigate possible catastrophic expenditures. They also need to take into account the perspective of the carers and full information on all opportunity costs incurred by taking up care giving activities and the burden it may be generating, such as negative health effects.

In addition, given the economic burden on caregivers with patients at the IMW, the difference in indirect costs between the two types of services is considerable. This figure is also relevant given that IMSS has only one GEMU. Thus, costs to caregivers could be expected to be as high in these other units. From the results of the model estimations we can observe the relevance that type of service has in determining type of OOPE and level of expenditures. This data could also be useful for policy makers that face a growing demand of older population and their specific needs in order to include health services models that respond in a comprehensive way to clinical, economic and social aspects.

Studies in Mexico as in other countries [14,30,31] have showed that the intensity and frequency of care needed by older adults is undertaken by their partners, children, friends, or other siblings, all of them non-remunerated. Notwithstanding, health system institutions and studies of health care systems usually ignore that informal care represents an essential piece of health care and as such should be taken into account. In light of the accelerated increase in the ageing population in the country, policy makers need to urgently address the issue of unpaid, informal care for older adults. It is necessary to recognise its role and that it is thoroughly investigated at the patient, family-household, and societal level and most importantly, its impact on all these angles.

In this sample, the vast majority of caregivers were women who probably assume many other competing responsibilities. Thus, opportunity costs of informal care should also be systematically researched and the results used in the planning of strategies to support these caregivers [32].

It is interesting to see that even when older adults in the study were admitted to hospital and provided the acute health care needed; the vast majority required an informal caregiver to provide additional support. In the future, studies on informal care giving should include in-depth qualitative studies in order to investigate further about the care the institutions are asking them to provide, by type of activity, the frequency, and intensity of care required.

In planning for new services or restructuring care for older adults in Mexico, institutions should also look into social values, beliefs, and economic consequences around having a family member or a loved one hospitalized. Although it has been noted how it is culturally expected to have family around the patient all the time, further studies should investigate how much having informal care in addition to all health care received responds to cultural values and how much it responds to institutions transferring the burden and the costs to the household. Health care planners and decision makers should be aware of this economic burden; the opportunity costs involved, and generate strategies to alleviate it [33].

Given the results using this data, further studies should investigate the differences in care provided for older adults at an IMW and GEMU in order to search for the best alternative and a more efficient way to provide acute care for this population group. Although one of the reasons that care is taken up by the family has to do with cultural traditions, an increasing cause has to do with hospitals discharging patients sooner and transferring health care activities to a primary caregiver at home and this should be further investigated [34]. One policy implication for Mexico is that in rationing decisions in health, many criteria like cost-effectiveness, equity and feasibility concerns play a major role. Relevant criteria have to be taken into account in order to make the best decision. In this report, an evaluation of health expenditures in a specific health care services setting, the GEMU, is added to a clinical evaluation previously reported [12]. It is hoped that both criteria will be considered in future planning and policy decisions, and in setting priorities of care. Furthermore, additional research looking for strong evidence has to be run.

In the context of a rapidly ageing population, the results of this study add evidence to the fact that national level policies should recognise the value of informal care and how it is expected to change in the future. There is a great need to generate policies that get together health and social care in order to provide long-term care services for older adults in order to provide them with optimal care, reduce expenditures, hospital admissions and readmissions, and ultimately, relieve the families from an important part of this burden of care.

Finally, there are some limitations to the data on carers used in this study. Additional information on their occupation, education level, the type of care activity provided, and the total number of hours caring would be desirable in order to generate further, more detailed analyses. Although we did not have additional information on these characteristics, one could expect these carers, mostly women, to have many other competing responsibilities such as domestic activities, caring for children and grandchildren, work outside home, among others, with scarce or no support to aide them in these difficult tasks. Moreover, some authors assume that an economic burden is also represented by domestic lost work by these women [35]. Qualitative information through focus groups and in-depth interviews in order to obtain more information on patient accompaniment values would have been desirable and should be included in future studies in order to have more complete information for the planning and decision making process regarding acute care for older adults.

## Conclusions

This study allowed us to identify economic burden related to acute care of elderly; in particular expenses often overlooked, potentially impacting the family, and should alert stakeholders from different sectors to open an agenda on this issue in order to reframe on going public policies or start new ones that give response to these unmet needs of a growing sector of the population. It also gives light to the urgent need to have more studies on informal care for older adults in order to properly inform such policies.

Although the data is restricted to a social security institution, the IMSS, it provides services for approximately 35% of total population in the country and we could say this study can represent a relevant portion of total population. In addition, this study adds to the scarce literature in Latin America on OOPE and diverse factors of informal care giving for older adults. This can be used to inform older adult care policies and generate further information as to design schemes that support informal carers and allows older adults to have the best care possible, reduce hospitalisations, etc.

## Competing interests

No author of this paper has a conflict of interest, including specific financial interests, relationships, and/or affiliations relevant to the subject matter included in this manuscript.

## Authors’ contributions

MLO made important contributions in the conception, design, acquisition and interpretation of data, drafted the manuscript, performed data analysis; VGG and JJGG were involved in critically revising the manuscript and performed part of the analysis; CGP had the original idea, made important contributions in the conception, design, acquisition and interpretation of data MUPZ made important contributions in the conception, design, acquisition and interpretation of data, drafted the manuscript. All authors approved the final version of the manuscript.

## Pre-publication history

The pre-publication history for this paper can be accessed here:

http://www.biomedcentral.com/1472-6963/13/51/prepub

## Supplementary Material

Additional file 1: Annex 1 Glossary of terms. Click here for file
