# Supplementary material for: Economic burden to primary informal caregivers of hospitalized older adults in Mexico: a cohort study
Source: BMC Health Serv Res. 2013 Feb 8;13:51. doi: 10.1186/1472-6963-13-51 (PMC3610123; doi:10.1186/1472-6963-13-51)
Supplement: Additional file 1: Annex 1 — Glossary of terms. [file 1472-6963-13-51-S1.docx]

**Annex 1**

**Glossary of Terms**

| **Key Variables** | **Definition** | **Score** | **Meaning of the Score** | **Hypothesis** |
| --- | --- | --- | --- | --- |
| Barthel Index  Functional Status (or activities of daily living) (Roy et al. 1988) | The Barthel Index consists of 10 items that measure a person's daily functioning specifically the activities of daily living and mobility. The items include feeding, moving from wheelchair to bed and return, grooming, transferring to and from a toilet, bathing, walking on level surface, going up and down stairs, dressing, continence of bowels and bladder. A validated Spanish version of the Barthel Index was used. | Same scoring as in the original scale was used (range 0 thru 100). | The highest score corresponding to the best possible functional subject in activities of daily living | Higher scores are associated with low OOPE and indirect costs, because the subject would require less help |
| Charlson Index (Charlson et al. 1987) | Comorbidity was evaluated using the Charlson Index.  The Chalrson Comorbidity Index is a prognostic Index proposed Mary Charlson et al in 1987. Participants were asked whether they had a diagnosis of 19 chronic diseases according the World Health Organization’s International Classification of Diseases, ICD-10 (WHO 2004). | Each disease was scored from 1 to 6 according the risk of death. Severity of comorbidity was established under a total score | 1 = not severe; 2 = mild; 3 = moderate and 4 = severe. | Higher scores would be associated with higher OOPE and indirect costs, because a sicker person has a higher probability of requiring drugs or studies because of chronic conditions in which the caregiver may require to pay for them |
| Mini Mental State Examination (MMSE)  (Folstein et al.1975) | Cognitive function was assessed using a validated Spanish version of the Folstein Mini-Mental State Examination (MMSE). The MMSE evaluates memory, orientation to space and time, calculation, language and word recognition, | Scores range from 0 to 30 points with lower scores indicating poorer cognitive ability. | Cognitive impairment was determined using a cutoff point of ≤ 23, adjusted by age. | Higher scores would be associated with lower OOPE and indirect costs, because subjects with cognitive impairment (lower scores) could need more help, specialized mental care and continuous presence of caregiver due to a higher probability of incident delirium which requires close intervention of the caregiver in order to prevent it or alleviate it |
| Acute Physiology and Chronic Health Evaluation II (APACHE II) score (Knaus et al.,1985) | APACHE II is a reliable tool to assess the severity of an acute condition, and integrates a series of laboratory data (i.e. sodium), vital signs (i.e. heart rate) and clinical items (neurological status). | Maximum score of 76 points, corresponding to a 99.9% hospital mortality rate | The higher the score the higher the probability the subject could die in a short term because of the acute illness that took him to the hospital | Higher scores are associated with higher OOPE and indirect costs, due to a higher probability of need of specialized critical care attention, expensive drugs or studies not usually available ; in addition to a required continuous presence of the caregiver in order to be available in case of any needs that rise during care |
| Satisfaction with Health Care Received, Client Satisfaction Questionnaire (CSQ 8) first question (Larsen et al. 1979) | Perception with the quality of health care received and expressed as satisfaction was assessed with the first item of the Client Satisfaction Questionnaire (CSQ 8) (Larsen et al. 1979); in which the subject is asked: “How would you rate the quality of the care you received during this hospitalization?” | Answer was dichotomized in Good or Average/Bad | Due to the dichotomized and mutually exclusive answer the interpretation is straightforward; satisfied with services received (Good) and not satisfied with services received (Average/Bad) | A satisfied patient and caregiver could have lower OOPE and indirect costs, and viceversa; because of the perception that all the needs of the ill elderly are already fulfilled and no need for other expenses in order to compensate unmet needs would be required. |
